# Immobilizing Polyether Imidazole Ionic Liquids on ZSM-5 Zeolite for the Catalytic Synthesis of Propylene Carbonate from Carbon Dioxide

**DOI:** 10.3390/molecules23102710

**Published:** 2018-10-21

**Authors:** Liying Guo, Xianchao Jin, Xin Wang, Longzhu Yin, Yirong Wang, Ying-Wei Yang

**Affiliations:** 1 School of Petrochemical Engineering, Shenyang University of Technology, Liaoyang 111003, China; xcjin1993@163.com (X.J.); yinlz18@mails.jlu.edu.cn (L.Y.); yrwang2000@163.com (Y.W.); 2 State Key Laboratory of Inorganic Synthesis and Preparative Chemistry, International Joint Research Laboratory of Nano-Micro Architecture Chemistry (NMAC), College of Chemistry, Jilin University, 2699 Qianjin Street, Changchun 130012, China; xinwangjlu@163.com

**Keywords:** catalyst, CO_2_, heterogeneous catalysis, molecular sieve, polyether imidazole ionic liquid

## Abstract

Traditional ionic liquids (ILs) catalysts suffer from the difficulty of product purification and can only be used in homogeneous catalytic systems. In this work, by reacting ILs with co-catalyst (ZnBr_2_), we successfully converted three polyether imidazole ionic liquids (PIILs), i.e., HO-[Poly-epichlorohydrin-methimidazole]Cl (HO-[PECH-MIM]Cl), HOOC-[Poly-epichlorohydrin-methimidazole]Cl (HOOC-[PECH-MIM]Cl), and H_2_N-[Poly-epichlorohydrin-methimidazole]Cl (H_2_N-[PECH-MIM]Cl), to three composite PIIL materials, which were further immobilized on ZSM-5 zeolite by chemical bonding to result in three immobilized catalysts, namely ZSM-5-HO-[PECH-MIM]Cl/[ZnBr_2_], ZSM-5-HOOC-[PECH-MIM]Cl/[ZnBr_2_], and ZSM-5-H_2_N-[PECH-MIM]Cl/[ZnBr_2_]. Their structures, thermal stabilities, and morphologies were fully characterized by Fourier-transform infrared spectroscopy (FT-IR), X-ray diffractometry (XRD), thermogravimetric analysis (TGA), and scanning electron microscopy (SEM). The amount of composite PIIL immobilized on ZSM-5 was determined by elemental analysis. Catalytic performance of the immobilized catalysts was evaluated through the catalytic synthesis of propylene carbonate (PC) from CO_2_ and propylene oxide (PO). Influences of reaction temperature, time, and pressure on catalytic performance were investigated through the orthogonal test, and the effect of catalyst circulation was also studied. Under an optimal reaction condition (130 °C, 2.5 MPa, 0.75 h), the composite catalyst, ZSM-5-HOOC- [PECH-MIM]Cl/[ZnBr_2_], exhibited the best catalytic activity with a conversion rate of 98.3% and selectivity of 97.4%. Significantly, the immobilized catalyst could still maintain high heterogeneous catalytic activity even after being reused for eight cycles.

## 1. Introduction

Carbon dioxide (CO_2_) is a rich carbon resource in nature. Over the years, experts and scholars around the world have been working on the catalytic conversion and utilization of CO_2_ [1,2,3,4,5]. Although research results have shown that the chemically speaking CO_2_ is extremely inactive, careful selection of proper catalysts could make CO_2_ become a low-cost and widely used resource. Therefore, the development of efficient catalysts is the key to achieve chemical fixation and conversion of CO_2_ under mild conditions.

Ionic liquids (ILs), as environmentally friendly chemical materials, have been widely used in various fields including organic synthesis, biomass dissolution, catalysis, and composite materials preparation due to their unique properties [6,7,8,9,10]. In recent years, studies on the conversion of CO_2_ to cyclic carbonate using ILs as catalysts have been widely reported [11,12,13], demonstrating that the catalytic performance of ILs as a single component in catalysts is unfavorable, and the addition of Lewis acids or other co-catalysts in the catalysts is necessary to achieve better catalytic activity. However, these studies are still associated with several disadvantages including limitation of the intermittent tank reaction system, catalyst separation, and product purification [14]. These defects make ILs difficult to be applied in industrial processes. To solve these problems, a structural design concept based on ILs-immobilized catalysts has gained considerable attention. Yin and coworkers [15] immobilized 3-(2-hydroxyethyl)-1-propylimidazolium bromide ILs on SBA-15, Al-SBA-15, and SiO_2_, but the yield declined obviously after three cycles of catalysis. Zhang’s group [16] and Xiong‘s group [17] immobilized ILs on chitosan and coconut shell activated carbon supporter, respectively. However, gradual loss of active components in the immobilized catalysts also occurred in the catalytic process. Therefore the utilization of chemical bonding between ILs and supporters is key to realize the resource transformation of CO_2_. Compared with other catalytic systems, such as metal [18], metal complexes [19], and imidazolium salt [20], ILs with the advantages of low energy consumption, high recycling efficiency, and less corrosion to equipment have become a hotspot [16]. Polyether polymer catalysts contain macromolecular chains that can form a synergistic and base barrier effect to enhance catalytic activity [21].

**Figure 1:**
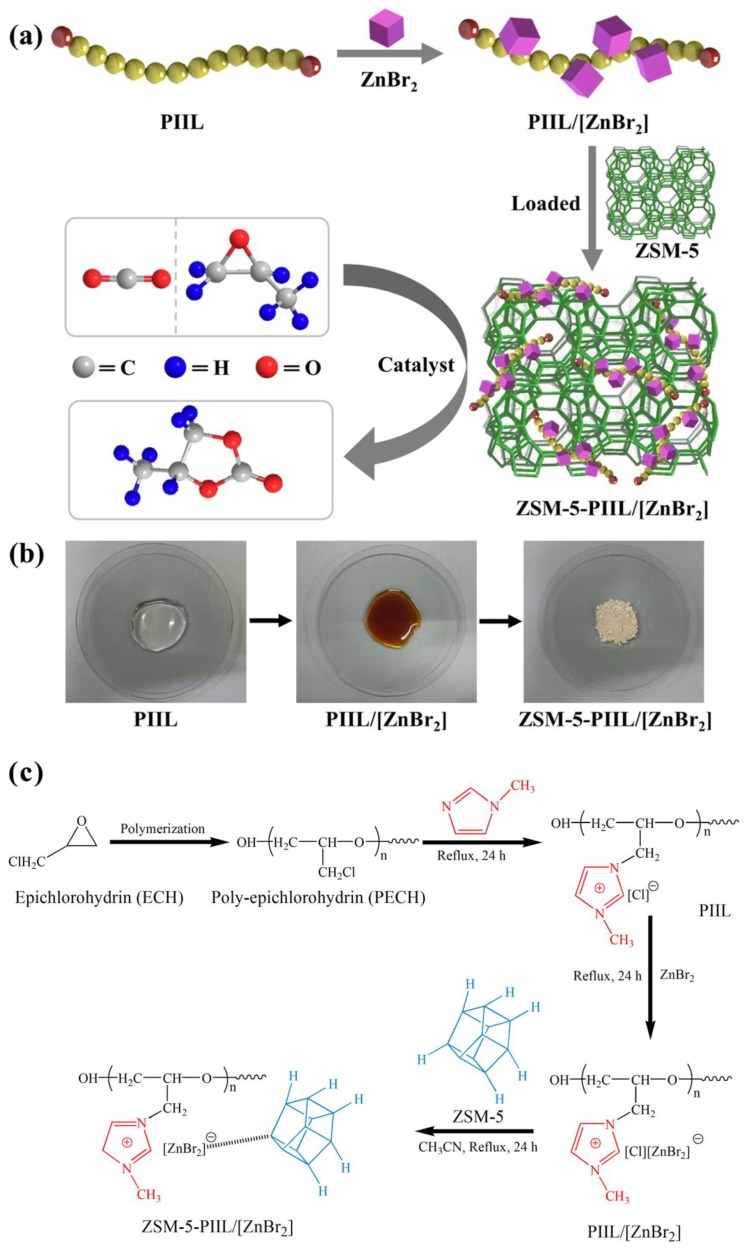
(**a**) The diagram for the preparation of immobilized catalysts for the synthesis of propylene carbonates from carbon dioxide; (**b**) the photos of the synthesized materials showing the color change and phase transition; and (**c**) the chemical structure of the catalyst and its synthesis process, taking ZSM-5-HO-[PECH-MIM]Cl/[ZnBr_2_] as an example.

In this work, three polyether imidazole ionic liquids (PIILs) were selected to react with co-catalyst (ZnBr_2_) to obtain three composite PIILs, followed by further immobilization on ZSM-5 zeolite to result in three immobilized PIILs catalysts (Figure 1 and Supplementary Materials). The chemical bonding between the composite PIILs and ZSM-5 was determined and the phase transitions of PIILs were also achieved. The immobilization is stable, so that the prepared catalyst can still maintain high catalytic activity after eight cycles.

## 2. Results and Discussion

### 2.1. Fourier-Transform Infrared Spectroscopy (FT-IR)

**Figure 2:**
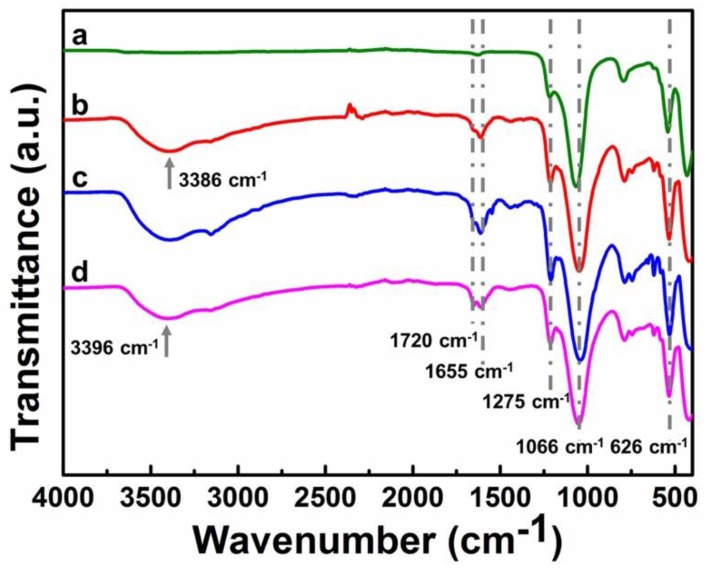
FT-IR spectra of zeolite ZSM-5 and its immobilized catalysts: (**a**) ZSM-5; (**b**) ZSM-5-HO-[PECH-MIM]Cl/[ZnBr_2_]; (**c**) ZSM-5-HOOC-[PECH-MIM]Cl/[ZnBr_2_]; and (**d**) ZSM-5-H_2_N-[PECH-MIM]Cl/[ZnBr_2_].

FT-IR studies on zeolite ZSM-5 and its immobilized catalysts were carried out (Figure 2). As in pattern (a), the peak at 1066 cm^−1^ corresponds to the Si–O characteristic peak of ZSM-5 [22]. Patterns (b, c, d) contain all the characteristic absorption peaks of pattern (a), and meanwhile, the characteristic peaks at ca. 1655 cm^−1^ and 626 cm^−1^, which are associated with the stretching frequency of the imidazole ring, were also found. The peaks at ca. 1275 cm^−1^ in patterns (b, c, d) could be assigned to the enhanced stretching vibration of C–O–C. The strong absorption peak at 3386 cm^−1^ of pattern (b) corresponds to the stretching vibration of the hydroxyl group. When polyether imidazole molecular chains are linked to ZSM-5, hydrogen chloride is released [15,23,24]. However, a small portion of HOOC-[PECH-MIM]Cl/[ZnBr_2_] still exists as deduced from the peak at 1720 cm^−1^ with low intensity in pattern (c), which is due to the physical adsorption on the surface of ZSM-5. In the immobilization process of H_2_N-[PECH-MIM]Cl/[ZnBr_2_], there are hydrogen atoms on the surface and inside of the molecular sieve [25]. A part of hydrogen on ZSM-5 reacted with the chloride ions of H_2_N-[PECH-MIM]Cl/[ZnBr_2_] thus releasing hydrogen chloride, and the other part of the hydrogen easily forms a hydrogen bond with oxygen in the polyether chain [15,23]. The hydrogen bonds caused the appearance of a wide and strong hydroxyl peak at 3396 cm^−1^ in pattern (d), thus the low amino peak was covered. The above results indicated the successful anchorage of composite PIILs on ZSM-5.

### 2.2. Scanning Electron Microscopy (SEM)

**Figure 3:**
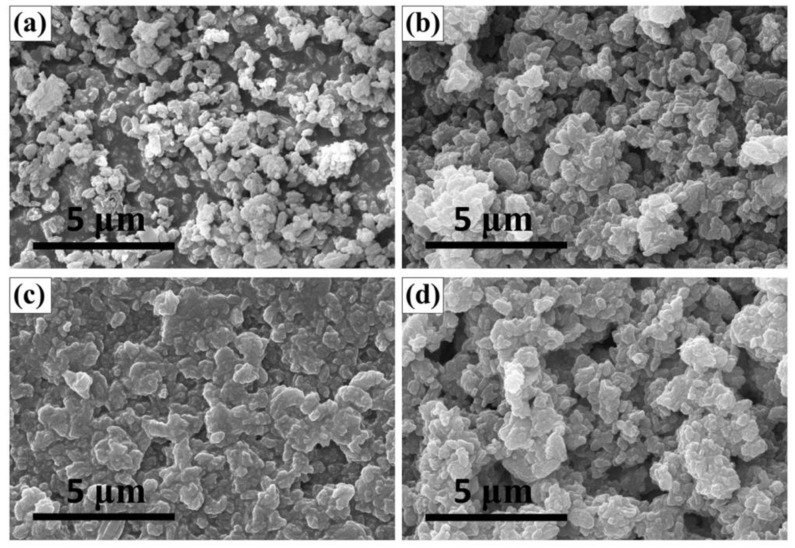
SEM images of zeolite ZSM-5 and its immobilized catalysts: (**a**) ZSM-5; (**b**) ZSM-5-HO-[PECH-MIM]Cl/[ZnBr_2_]; (**c**) ZSM-5-HOOC-[PECH-MIM]Cl/[ZnBr_2_]; and (**d**) ZSM-5-H_2_N-[PECH-MIM]Cl/[ZnBr_2_].

SEM images of ZSM-5 and its immobilized catalysts are provided in Figure 3. After the immobilization, the surfaces of the catalysts (b, c, d) were covered with a white translucent substance, and the surfaces became smoother. The particles became larger in size and their profiles became clearer, indicating that a part of the PIILs was immobilized on the surface of ZSM-5. SEM results further suggest the successful immobilization of PIILs catalysts on ZSM-5 and the solid state of the obtained immobilized catalysts. See Figure 1b for ordinary photos of the phase transition.

### 2.3. X-Ray Diffractometry (XRD)

**Figure 4:**
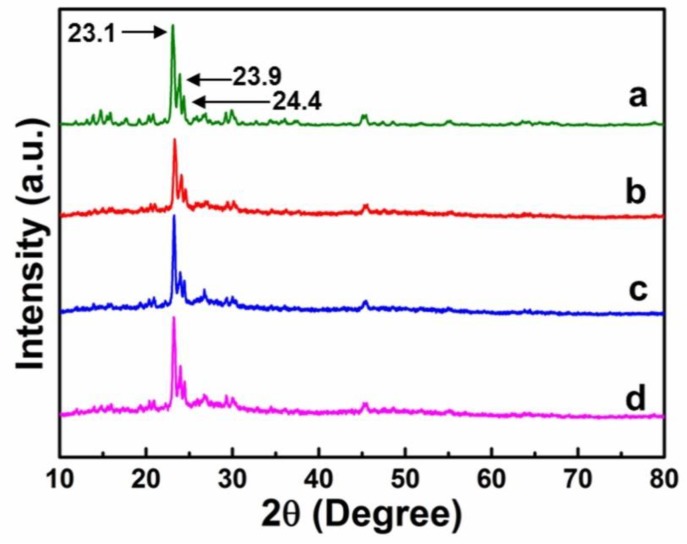
XRD patterns of zeolite ZSM-5 and its immobilized catalysts: (**a**) ZSM-5; (**b**) ZSM-5-HO-[PECH-MIM]Cl/[ZnBr_2_]; (**c**) ZSM-5-HOOC-[PECH-MIM]Cl/[ZnBr_2_]; and (**d**) ZSM-5-H_2_N-[PECH-MIM]Cl/[ZnBr_2_].

The XRD patterns of ZSM-5 and its immobilized catalysts are shown in Figure 4. In pattern (a), the peaks of high intensity at 23.1°, 23.9°, and 24.4° are the characteristic diffraction peaks of ZSM-5, indicating good crystallinity of our synthesized ZSM-5. Compared with pattern (a), patterns (b, c, d) exhibit all the diffraction peaks of ZSM-5, and the shape and intensity of the diffraction peaks have negligible changes, indicating that the prepared catalysts maintained the good crystallinity of ZSM-5 after immobilization of the three composite PIILs onto ZSM-5.

### 2.4. Thermogravimetry (TG) and Derivative Thermogravimetry (DTG)

**Figure 5:**
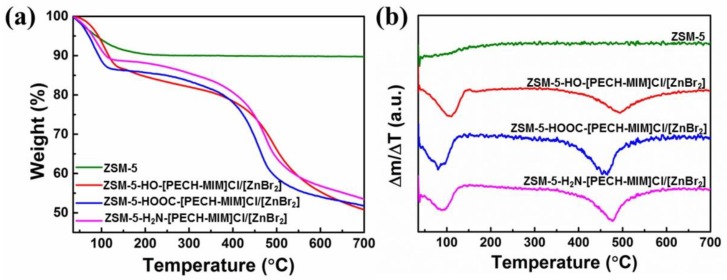
(**a**) TG and (**b**) DTG of zeolite ZSM-5 and its immobilized catalysts: ZSM-5-HO-[PECH-MIM]Cl/[ZnBr_2_]; ZSM-5-HOOC-[PECH-MIM]Cl/[ZnBr_2_]; and ZSM-5-H_2_N-[PECH-MIM]Cl/[ZnBr_2_].

Molecular sieve ZSM-5 and its immobilized catalysts were further characterized by thermogravimetric analyzer (TGA) to explore their thermal stability with a heating rate of 10 °C/min. TG and DTG results are shown in Figure 5. As seen in Figure 5a, ZSM-5 and the three catalysts have slight weight loss before 150 °C, which is caused by the evaporation of water. As the temperature rises, the curve of ZSM-5 in Figure 5b approximates to a straight line, where the weight loss rate does not change and ZSM-5 does not break down. From Figure 5a, the three immobilized catalysts were broken down in two steps. The first loss (230–550 °C) is attributed to the loss of grafted composite PIILs [26,27,28], and the second slight loss (550–700 °C) is due to the decomposition of some molecular sieve [29,30,31]. The three catalysts with functional groups of –OH, –COOH, and –NH_2_ have maximum decomposition rate at 490 °C, 460 °C, and 475 °C, respectively, and their residual rates are maintained above 50%. The decomposition speed and thermal stability of the three immobilized catalysts with different functional groups are different, and among them, ZSM-5-HOOC-[PECH-MIM]Cl/[ZnBr_2_] possesses the fastest decomposition and the highest residual rate. The thermal stability of the other two immobilized catalysts shows little difference, and the weight loss processes are similar. TG and DTG results suggest that the immobilized catalysts have good thermal stability, and that the immobilized catalysts would not decompose under 230 °C.

### 2.5. Effects of Different Catalysts on the Catalytic Performance

The catalytic performance of the above mentioned three PIILs, three composite PIILs, ZSM-5, and three immobilized catalysts for the synthesis of propylene carbonate (PC) from CO_2_ and propylene oxide (PO) was investigated (Table 1). Three PIILs showed catalytic activity (entries 1–3), among them, HOOC-[PECH-MIM]Cl has the best catalytic performance because the terminal carboxyl group has acidity and can also act as donor of a hydrogen bond, which could add a synergistic effect with the anion. The synergistic effect contributes to the activation and ring opening of PO and improves the catalytic activity [15,24]. Zinc bromide has a catalytic effect when used alone as a co-catalyst (entry 4). The catalytic performance of PIILs were further improved after addition of zinc bromide (entries 5–7) [26,27], where the conversion rate reached 100%, and the selectivity and yield both reached above 97%. The improvement of the catalytic performance can be attributed to the addition of zinc bromide that increased the amount of bromide ions. The Br^−^ attacked the β-carbon atom of PO, and therefore increased the conversion rate. The polyether macromolecular chain has a basic isolation effect and an infinite dilution effect, which could not only inhibit the formation of by-products but also improve the selectivity [32,33]. The catalytic activity of ZSM-5 is poor (entry 8). The excellent catalytic performance of the three immobilized catalysts is very promising, although there is no big difference between them (entries 9–11). This is because the structures of the immobilized catalysts were similar after three composite PIILs were immobilized on the molecular sieve by chemical bonding. The chemical bonding occurs through the covalent bond formation by condensation between the chlorine of PIILs and the hydrogen on ZSM-5 [25]. All of the three immobilized catalysts have polyether macromolecular chains as the main chains and imidazole rings as the branched chains. ZSM-5-HOOC-[PECH-MIM]Cl/[ZnBr_2_] has better catalytic activity than the other two immobilized catalysts, because there is still a small amount of HOOC-[PECH-MIM]Cl/[ZnBr_2_] physically adsorbed on the surface of ZSM-5. It still has acidity, which is good for ring opening [15,24], consistent with the FT-IR study. The catalytic activity of the immobilized catalysts is slightly lower than that of the composite PIILs since the amount of composite PIILs is reduced after immobilization. However, the conversion rate and selectivity both reached 95%. Experimental results showed an excellent catalytic performance of the three immobilized catalysts, among which ZSM-5-HOOC-[PECH-MIM]Cl/[ZnBr_2_] represents the best.

### 2.6. Effects of Reaction Conditions on the Catalytic Performance

The effect of reaction conditions on the catalytic performance of ZSM-5-HOOC-[PECH-MIM] Cl/[ZnBr_2_] was studied. To define the optimal reaction conditions, the orthogonal test of L9 (3^4^) was used to investigate the effects of reaction pressure, temperature, and time on the conversion rate and selectivity (Table 2). K_nj_ (n = 1,2,3) indicated the sums of three conversion rates or selectivity when the values of the corresponding factors were the same for each time. R was the range. The results show that reaction pressure has the greatest effect on conversion rate and selectivity, followed by reaction temperature and time. When the reaction pressure is 2.5 MPa, the temperature is 130 °C, and the time is 0.75 h, the immobilized catalyst exhibited the best catalytic performance with a conversion rate of 98.3% and a selectivity of 97.4%. When the reaction pressure is increased, more CO_2_ can be dissolved in PO, therefore, the conversion rate and selectivity can be increased. If the pressure was too high or the reaction time prolonged, the selectivity declined, which is consistent with the previous literature reports [16,34,35,36]. Excessive temperature could cause a decrease in selectivity due to the exothermic nature of the cycloaddition reaction that could lead to the production of some byproduct such as from the polymerization of PC [15,37].

### 2.7. Effect of the Catalyst Circulation on the Catalytic Performance

**Figure 6:**
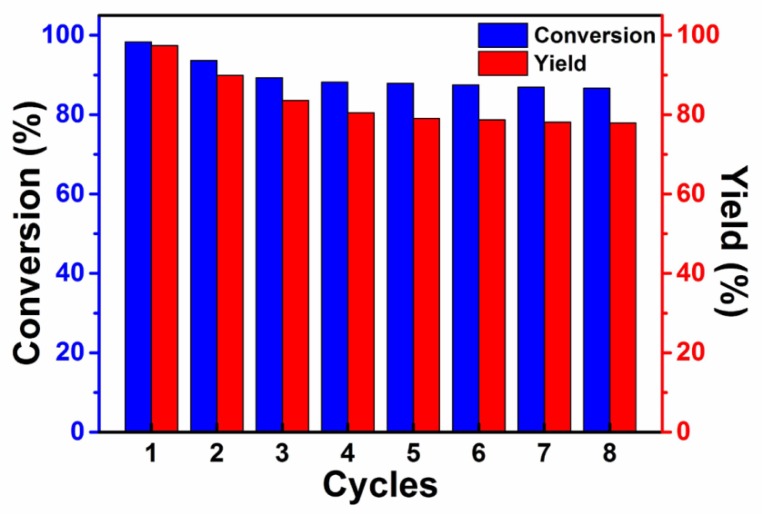
Effect of the catalyst circulation on catalytic performance.

The effect of catalyst circulation on catalytic performance was investigated under an optimized reaction condition (2.5 MPa, 130 °C, 0.75 h), with ZSM-5-HOOC-[PECH-MIM]Cl/[ZnBr_2_] as the catalyst. As in Figure 6, the experimental results suggest an excellent reusability of the immobilized catalyst. The conversion rate and yield are 98.3% and 97.4%, respectively, in the first use. In the next two cycles, the catalytic performance decreased. Elemental analysis was used to measure the fresh catalyst, three and eight cycle catalysts, respectively (Table 3). The results showed that the grafted rate of composite PIILs catalyst decreased over 1 to 3 cycles, and there was negligible change after three cycles. The reason is that a small amount of physically adsorbed PIILs is easily falls off from ZSM-5 zeolite during the 1st to 3rd cycles. From the 4th cycle, the grafted rate of PIILs, conversion rate and yield showed small changes, which indicate that the chemical bonding between the composite PIILs and the zeolite is steady, and therefore the catalytic performance of the immobilized catalyst remains stable. The conversion rate is 86.7% and the yield is 77.9% after the 8th cycle. The results in Figure 6 are consistent with those in Table 3. The amount of the grafted PIIL is 1.121 mmol/g, the chemical graft is about 0.803 mmol/g, and physical graft is about 0.318 mmol/g.

## 3. Materials and Methods

### 3.1. Reagents and Instruments

Three PIILs, i.e., HO-[PECH-MIM]Cl, HOOC-[PECH-MIM]Cl, and H_2_N-[PECH-MIM]Cl, were prepared according to our published procedures [26,27,28]. Zinc bromide was purchased from Tianjin Guangfu chemical reagents factory (Tianjin, China). Acetonitrile was received from Tianjin Guangfu Fine Chemical Research Institute and used without further purification. PO was obtained from Jiangsu Yonghua fine chemicals Research Company (Suzhou, China). ZSM-5 zeolite, CO_2_, and nitrogen were supplied by Liaoyang Petrochemical Industries Company (Liaoning, China).

Instruments used in this study included a MAGNA-IR750 Fourier Transform Infrared Spectrometer (FT-IR, Thermo Nicolet Corporation, Markham, ON, Canada); TM3000 Scanning Electron Microscope (SEM, Keyence, Osaka, Japan); D/max-2400 Automatic X-ray Diffractometer (XRD, RKC Instrument Inc., Tokyo, Japan); TGA4000 Thermogravimetric Analyzer (TGA, PerkinElmer, Waltham, MA, USA); PerkinElmer-2400 Element Analyzer (EA, PerkinElmer, Waltham, MA, USA); PARR4523 Catalytic Device (PARR, Moline, IL, USA); 1790F Gas Chromatograph (GC) (Agilent Technologies, Inc., Santa Clara, CA, USA); D08-8C Carbon Dioxide Flowmeter (Beijing Sevenstar Electronics Co., Ltd., Beijing, China); DF-101S Magnetic Stirrer (Gongyi Yuhua Instrument Co., Ltd., Gongyi, China); SFX-2L Rotary Evaporator (Taiwan Xinyue Instrument and Meter Co., Ltd., Xiamen, China); DZF-6050 Vacuum Drying box (Gongyi Yuhua Instrument Co., Ltd., Gongyi, China); 2-XZ-4 rotary vane vacuum pump (Wenling City Yangan Electromechanical Co., Ltd., Wenling, China).

### 3.2. Preparation of Immobilized Catalysts

Three PIILs, i.e., HO-[PECH-MIM]Cl, HOOC-[PECH-MIM]Cl, and H_2_N-[PECH-MIM]Cl, were prepared according to our published procedures [26,27,28]. Briefly, epichlorohydrin (ECH) was polymerized to form poly-epichlorohydrin (PECH) possessing –OH groups, then chloroacetic acid and ammonia were used to react with PECH to obtain products with –COOH and –NH_2_ groups, respectively. Then three types of PECH samples with –OH, –COOH, and –NH_2_ were reacted with imidazole to obtain three PIILs. Next, a given amount of ZnBr_2_ was added into the three PIILs, respectively, and the resulting mixtures were heated at reflux under stirring for 24 h. As the reaction proceeded, the mixtures became more viscous with a dark color change. When the reactions were complete, three reddish brown liquids were obtained, namely three composite PIILs, i.e., HO-[PECH-MIM]Cl/[ZnBr_2_], HOOC-[PECH-MIM]Cl/[ZnBr_2_], and H_2_N-[PECH-MIM]Cl/[ZnBr_2_]. Then, the composite PIILs were added, respectively, to the suspension of ZSM-5 in acetonitrile, and the resulting mixtures were stirred at 70 °C for 24–36 h to obtain milk-white liquids. Acetonitrile was removed from the obtained liquids by rotary evaporation at 65 °C. The afforded three samples of white powder were then dried in vacuum at 65 °C for 24 h under 0.08 MPa to remove residual acetonitrile, leading to the final products of immobilized PIILs catalysts, i.e., ZSM-5-HO-[PECH-MIM]Cl/[ZnBr_2_], ZSM-5-HOOC-[PECH-MIM]Cl/[ZnBr_2_] and, ZSM-5-H_2_N-[PECH-MIM]Cl/[ZnBr_2_] ( Figure 1c).

### 3.3. Typical Procedure for the Synthesis of PC from PO and CO_2_

The immobilized catalyst (2.5% mass fraction of PO) was added in the stainless-steel autoclave. After the atmosphere was replaced by nitrogen, PO and CO_2_ were filled into the autoclave. When the flow of CO_2_ was 0, the reaction was complete. The crude product was purified to obtain the refined PC, and its purity was determined by gas chromatography. Specific steps refer to our reported papers [26,27].

## 4. Conclusions

Three immobilized PIILs catalysts were newly developed, and their catalytic performance studied. The results showed that the immobilization of composite PIILs on ZSM-5 was mainly based on chemical bonding, while a small amount of physical adsorption also existed. ZSM-5-HOOC-[PECH-MIM]Cl/[ZnBr_2_] exhibited the highest catalytic activity in the synthesis of PC under the optimized condition: reaction temperature of 130 °C, pressure of 2.5 MPa, and time of 0.75 h, where the conversion rate reached 98.3% and the selectivity 97.4%. Significantly, the catalyst still maintained a good catalytic activity after eight cycles. We envisage that the newly prepared catalysts can solve the problems of traditional ILs catalysts including the difficulty in production purification and short service life in the catalytic process. We believe that our research can provide industrial continuous conversion of CO_2_ in a packed bed reactor with acceptable performance.

## Figures and Tables

**Table 1:** Effects of different catalysts on catalytic performance ^1^.

	Catalyst	Conversion Rate (%)	Selectivity (%)	Yield (%)	Purity (%)
1	HO-[PECH-MIM]Cl	90.4	96.4	87.1	99.1
2	HOOC-[PECH-MIM]Cl	95.1	96.8	92.1	99.4
3	H_2_N-[PECH-MIM]Cl	93.3	95.3	88.9	98.9
4	ZnBr_2_	40.8	68.2	27.8	95.2
5	HO-[PECH-MIM]Cl/[ZnBr_2_]	100	97.1	97.1	99.5
6	HOOC-[PECH-MIM]Cl/[ZnBr_2_]	100	98.9	98.9	99.7
7	H_2_N-[PECH-MIM]Cl/[ZnBr_2_]	100	97.6	97.6	99.4
8	ZSM-5	9.4	41.2	3.87	96.4
9	ZSM-5-HO-[PECH-MIM]Cl/[ZnBr_2_]	95.8	96.5	92.4	98.3
10	ZSM-5-HOOC-[PECH-MIM]Cl/[ZnBr_2_]	96.3	96.8	93.2	99.2
11	ZSM-5-H_2_N-[PECH-MIM]Cl/[ZnBr_2_]	96.1	96.4	92.6	99.0

^1^ Reaction condition: catalyst, 2.5 wt%, temperature, 120 °C; pressure, 2.5 MPa; time, 1 h.

**Table 2:** Orthogonal test-effects of reaction conditions on catalytic performance.

	Factor			Conversion Rate (%)	Selectivity (%)
	Pressure (MPa)	Temperature (°C)	Time (h)		
1	2.0	110	0.75	82.3	92.4
2	2.0	120	1.0	90.6	90.1
3	2.0	130	1.25	95.4	85.2
4	2.5	110	1.0	95.9	98.2
5	2.5	120	1.25	97.6	97.5
6	2.5	130	0.75	98.3	97.4
7	3.0	110	1.25	92.1	94.6
8	3.0	120	0.75	97.9	90.3
9	3.0	130	1.0	99.5	84.2
K_1j_	268.3/267.7	270.3/285.2	278.5/280.1	—	—
K_2j_	291.8/293.1	286.1/277.9	286.0/272.5	—	—
K_3j_	289.5/269.1	293.2/266.8	285.1/277.3	—	—
R	23.5/25.4	22.9/18.4	7.5/7.6	—	—

**Table 3:** Elemental analysis results of the catalysts.

Catalyst	N (wt%)	C (wt%)	H (wt%)	PIILgrafted (mmol/g)	Standard Error (mmol/g)
ZSM-5-HOOC-[PECH-MIM]Cl/[ZnBr_2_] ^1^	3.149	7.744	2.000	1.121	1.780 × 10^−3^
ZSM-5-HOOC-[PECH-MIM]Cl/[ZnBr_2_] ^2^	2.275	7.131	1.694	0.810	1.472 × 10^−3^
ZSM-5-HOOC-[PECH-MIM]Cl/[ZnBr_2_] ^3^	2.256	7.059	1.681	0.803	1.633 × 10^−3^

^1^ Fresh catalyst; ^2^ Catalyst reused 2 times; ^3^ Catalyst reused 8 times.

## Supplementary Materials

The supplementary materials are available online.
