# Supplementary material for: Immobilizing Polyether Imidazole Ionic Liquids on ZSM-5 Zeolite for the Catalytic Synthesis of Propylene Carbonate from Carbon Dioxide
Source: Molecules. 2018 Oct 21;23(10):2710. doi: 10.3390/molecules23102710 (PMC6222835; doi:10.3390/molecules23102710)
Supplement: Supplementary file 1 [file molecules-23-02710-s001.pdf]

Supplementary Materials

Immobilizing Polyether Imidazole Ionic Liquids on ZSM-5 Zeolite for the Catalytic Synthesis of Propylene Carbonate from Carbon Dioxide

Liying Guo [1,*], Xianchao Jin [1], Xin Wang [2], Longzhu Yin [1,2], Yirong Wang [1] and Ying-Wei Yang [2,*]

[1] School of Petrochemical Engineering, Shenyang University of Technology, Liaoyang 111003, China

[2] State Key Laboratory of Inorganic Synthesis and Preparative Chemistry, International Joint Research Laboratory of Nano-Micro Architecture Chemistry (NMAC), College of Chemistry, Jilin University, 2699 Qianjin Street, Changchun 130012, China

xcjin1993@163.com (X.J.); xinwangjlu@163.com (X.W.); yinlz18@mails.jlu.edu.cn (L.Y.); yrwang2000@163.com (Y.W.)

* Correspondence: lyguo1981@163.com (L.G.); ywyang@jlu.edu.cn (Y.-W.Y.); Tel.: +8615140998399 (L.G.); +86-431-8516-8468 (Y.-W.Y.)

1. The synthesis of PIILs[S1-S3]

1.1. Preparation of HO-[PECH-MIM]Cl

1, 2-Dichloroethane (19.6 mL), ethylene glycol (2.8 mL) and boron trifluoride etherate (2 mL) were added into a flask equipped with a thermometer, stirrer, and condensing tube. N_2 was purged into the system to replace the air inside. The mixture was stirred at room temperature. After stirring for 30 min, the temperature of the reaction system was reduced to 0 °C in an ice bath, and then epichlorohydrin (ECH) (39.2 mL) and 1, 2-dichloroethane (15 mL) were mixed and slowly added to the reaction system. After 3 h the solution became a colorless and viscous liquid. Then, the reaction mixture was transferred to a separatory funnel and washed with distilled water for three times, and then transferred to a distillation flask and distilled under the pressure of 0.09 MPa at 70 °C for 3 h to remove low-boiling solvents and organics, resulting in a refined polyepichlorohydrin (PECH). Toluene (50 ml) and PECH (40 g) were added into a round-bottom flask. After stirring for 8 min, N-methylimidazole (65 ml) was added to the mixture. The mixture was stirred at 50 °C for 10 h and then cooled to room temperature, followed by the addition of ether (50 ml). The reaction mixture was transferred to a distillation flask and distilled under the pressure of 0.1 MPa at 90 °C to remove the solvent and low-boiling organic matters to obtain the polyether imidazole ionic liquid (PIIL), namely HO-[PECH-MIM]Cl.

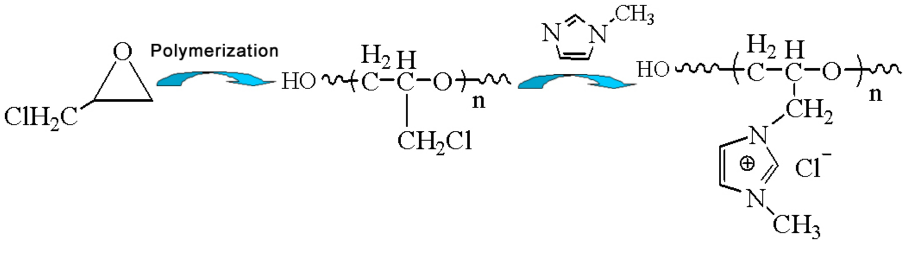

Figure S1. Synthetic route of HO-[PECH-MIM]Cl.

1.2. Preparation of HOOC-[PECH-MIM]Cl

PECH (8.2 g) and chloroacetic acid (18 g) were added into a flask equipped with a condenser tube and added acetonitrile (50 ml). The mixture was stirred at 80 °C for 1 h and then Na_2CO_3 (4 g) was added into the system. After 6~8 h, the pH of the reaction system was adjusted to 3~4 by adding hydrochloric acid (7 mL), and acetonitrile was removed by rotary evaporation under the pressure of 0.09 MPa at 70 °C, and washed with distilled water for 3 to 5 times to remove a small amount of low molecular weight substances and excess chloroacetic acid. After that, a small amount of water was removed under the pressure of 0.09 MPa at 80 °C, HOOC-[PECH]Cl (8.4 g) was obtained. HOOC-[PECH]Cl (3.65 g) and N-methylimidazole (4.8 ml) were mixed in the flask, the mixture was stirred at 80 °C for 24 h. The product was washed three times with ethyl acetate and dried in a vacuum drying oven under the pressure of 0.09 MPa at 60 °C for 24 h to obtain the PIILs (5.36 g), namely HOOC-[PECH-MIM]Cl.

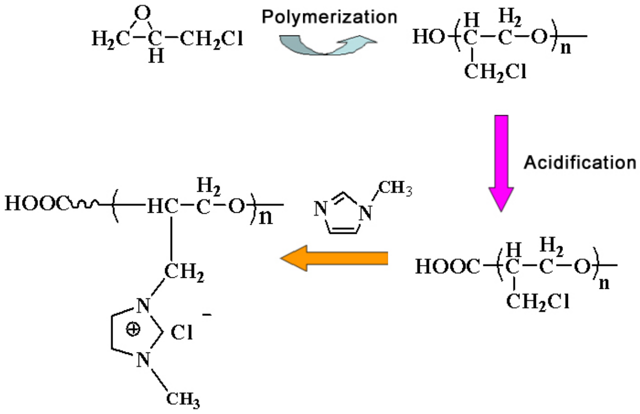

Figure S2. Synthetic route of HOOC-[PECH-MIM]Cl.

1.3. Preparation of H_2N-[PECH-MIM]Cl

A mixture of toluene and PECH in a mass ratio of 2:1 was added into a flask equipped with reflux condensation, and the mixture was stirred. The bromine was slowly added to the mixture in a separatory funnel. The reaction was continued at room temperature for 2 h after the addition was completed, and then the reaction was continued for 3 h by gradually increasing the temperature and maintaining the temperature (not exceeding 50 °C). Excessive ammonia was slowly added to the solution, and the reaction was continued until the color of the solution changed from reddish brown to colorless, the temperature was maintained at 30 °C, and after 3 h, the oily liquid was separated using a separatory funnel. Then the oily liquid was washed with deionized water for 5 times and then toluene and small molecular substances were removed on a rotary vap under the pressure of 0.09 MPa at 80 °C to obtain H_2N-[PECH]Cl. H_2N-[PECH]Cl and N-methylimidazole were added into the flask at a certain mass ratio, the mixture was stirred and reacted for 24 h at a constant temperature water bath at 80 °C. The resulting product was washed three times with ethyl acetate and placed in drying oven at 70 °C for 12 h to obtain the PIILs, namely H_2N-[PECH-MIM]Cl.

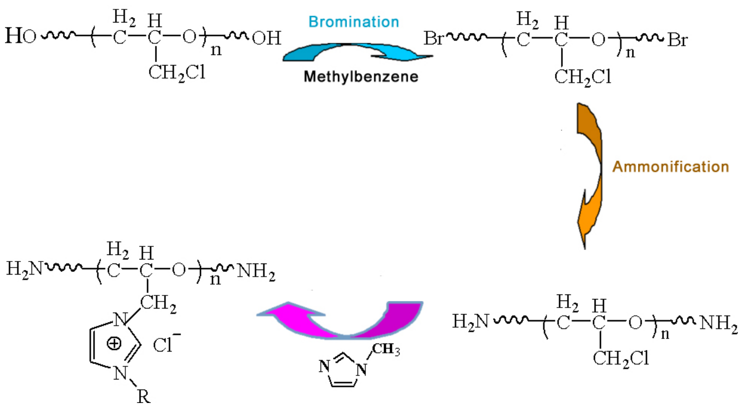

Figure S3. Synthetic route of H_2N-[PECH-MIM]Cl.

2. Typical Procedure for the Synthesis of PC from PO and CO_2

The catalyst (2.5% mass fraction of the PO) was added into autoclave (300 ml). Replaced internal air with nitrogen after sealed the reactor. CO_2 was filled in the autoclave through the intake bypass, when the pressure was 1.5 MPa, PO (150 ml) were added by a metering pump. The flow of CO_2 was regulated. The reaction temperature (130 °C), pressure (2.5MPa), and stirring rate (200 r/min) were set. Cooling water was turned on to cool the system to room temperature and the pressure was relieved when the flow of CO_2 was zero. To purify prepared PC, the product was added into distilled bottle for vacuum distillation under the condition of temperature (135°C) and pressure (0.09 MPa), the residual catalyst was reused. The obtained colorless liquid was PC. The PC was weighed and the purity was determined by gas chromatography.

3. ^{1}H NMR spectra of PIILs

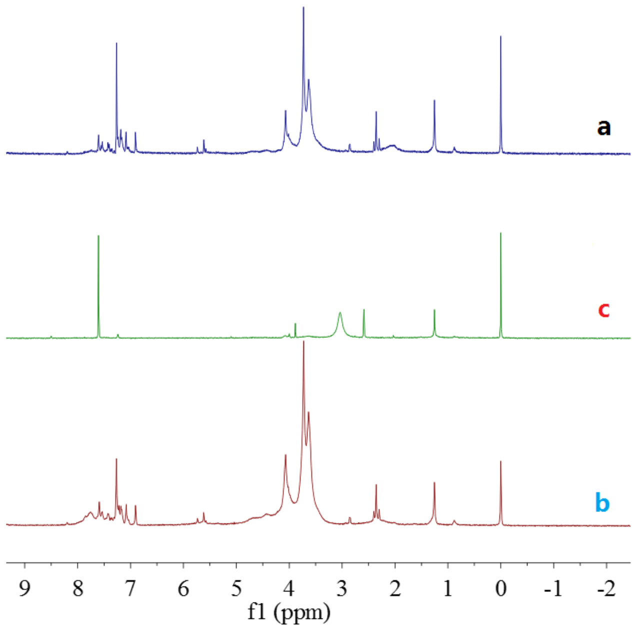

Figure S4. ^{1}H NMR spectra of three polymer ionic liquids: (a) [HO-PECH-MIM]Cl; (b) [H_2N-PECH-MIM]Cl; (c) [HOOC-PECH-MIM]Cl.

^{1}H NMR spectra of three catalysts were shown in Figure 1. In the spectrum a, δ=2.82 corresponds to the chemical shift of the active hydrogen on -OH, δ=3.88 corresponds to the chemical shift of the H atom of $-CH_3$ connected to N atom on imidazole ring, δ=7.47 corresponds to the chemical shift of the H atom of -CH which was connected to two N atoms on imidazole ring, δ=7.05 and δ=6.89 correspond to the chemical shifts of the H atom of $-CH_2$ and-CH, respectively, connected to N atoms on imidazole ring.

In the spectrum b, δ=4.48 corresponds to the chemical shift of the hydrogen on $-NH_2$, and the hydrogen is active H, δ=3.69 corresponds to the chemical shift of H atom on $-CH_3$ connected to N on imidazole ring, δ=7.9 corresponds to the chemical shift of H atom on $-CH_3$ connected to two N atoms on imidazole ring, and δ=7.2 and 7.0 corresponds to the chemical shifts of H atoms on $-CH_2$ and -CH of imidazole ring respectively.

In the spectrum c, δ=5.11 corresponds to the chemical shift of the active hydrogen at -COOH, δ=3.03 corresponds to the chemical shift of the hydrogen on N-linked $-CH_3$ of imidazole ring, δ=8.48 corresponds to the chemical shift of hydrogen on -CH of two N atoms on imidazole ring, δ=7.61 and 7.24 correspond to the chemical shift of hydrogen on $-CH_2$ of imidazole ring, δ=4.10 corresponds to the chemical shift of hydrogen on -CH_2 of polyether branched chain connected with imidazole ring, δ=3.86 corresponds to the chemical shift of hydrogen on $-CH_2$ in polyether main chain. The chemical shift of hydrogen on the -CH of the main chain is 3.98

4. TG results of PIILs

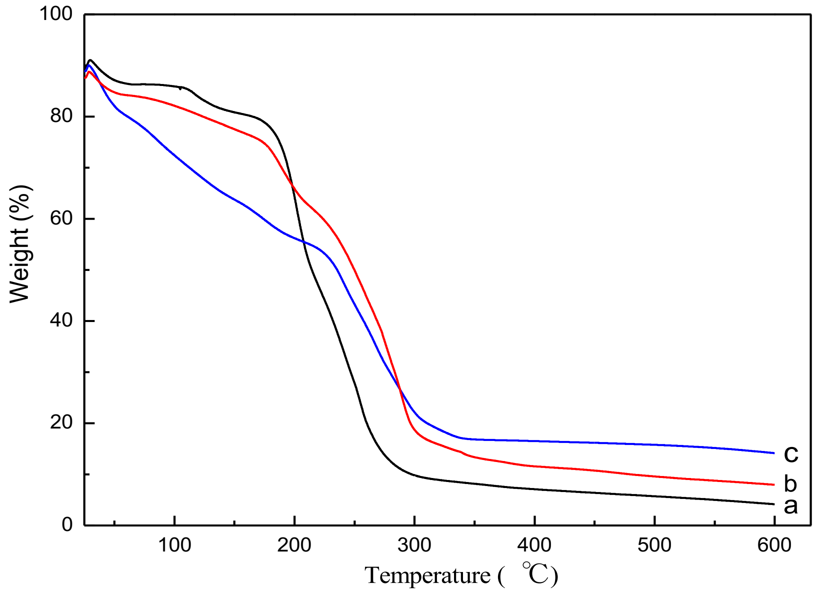

Figure S5. TGA curves of three polymer ionic liquids: (a) HO-[PECH-MIM]Cl; (b) H_2N-[PECH-MIM]Cl; (c) HOOC-[PECH-MIM]Cl.

From the TGA results of PIILs, the first weight loss is caused by the evaporation of water in PIILs from 50 to 220 °C, the second weight loss is due to the degradation of PIILs from 220 to 600 °C. Residual rates of HO-[PECH-MIM]Cl, H_2N-[PECH-MIM]Cl and HOOC-[PECH-MIM]Cl were 4.2%, 8.1% and 14.0% respectively.

5. Elemental analysis results of the ZSM-PIILs[$ZnBr_2$]

Table S1 Elemental analysis results of the catalysts (a)

Entry	Cycle times	N (wt%)	C (wt%)	H (wt%)	PIIL grafted (mmol/g)
1	1	3.146	7.743	1.997	1.120
	2	2.270	7.132	1.694	0.810
	8	2.256	7.059	1.682	0.800
2	1	3.150	7.747	2.002	1.121
	2	2.280	7.129	1.693	0.812
	8	2.252	7.061	1.680	0.802
3	1	3.143	7.740	1.993	1.119
	2	2.280	7.130	1.692	0.812
	8	2.258	7.061	1.683	0.804
4	1	3.151	7.749	1.992	1.122
	2	2.269	7.135	1.689	0.808
	8	2.260	7.063	1.685	0.805
5	1	3.157	7.745	2.010	1.124
	2	2.273	7.129	1.701	0.809
	8	2.252	7.058	1.679	0.802
6	1	3.144	7.737	2.008	1.119
	2	2.275	7.128	1.695	0.810
	8	2.255	7.053	1.675	0.803

(b)

Cycle times	Average PIIL grafted (mmol/g)	Standard error (mmol/g)
1	1.121	1.780×10^{-3}
2	0.810	1.472×10^{-3}
8	0.803	1.633×10^{-3}

(c)

Catalyst	N (wt%)	C (wt%)	H (wt%)	PIILgrafted (mmol/g)	Standard error (mmol/g)
ZSM-5-HOOC-[PECH-MIM]Cl/[$ZnBr_2$][1]	3.149	7.744	2.000	1.121	1.780×10^{-3}
ZSM-5-HOOC-[PECH-MIM]Cl/[$ZnBr_2$][2]	2.275	7.131	1.694	0.810	1.472×10^{-3}
ZSM-5-HOOC-[PECH-MIM]Cl/[$ZnBr_2$][3]	2.256	7.059	1.681	0.803	1.633×10^{-3}

6. Molecular weights of PIILs

Using tetrahydrofuran as solvent and molecular weight of polystyrene (PS) as the contrast, the molecular weights of three PIILs were determined by gel permeation chromatography (GPC). Figure 6 showed the mass distribution of three PIILs.

The molar weights of HO-[PECH-MIM]Cl, HOOC-[PECH-MIM]Cl and H_2N-[PECH-MIM]Cl were 1261, 1914 and 2286, respectively.

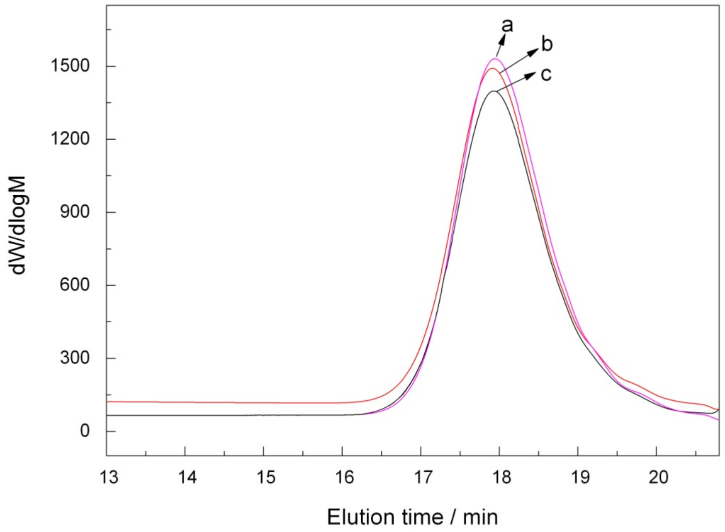

Figure S6. The GPC traces of PIILs: (a) H_2N-[PECH-MIM]Cl; (b) HOOC-[PECH-MIM]Cl; (c) HO-[PECH-MIM]Cl.

7. FT-IR spectra of PIILs

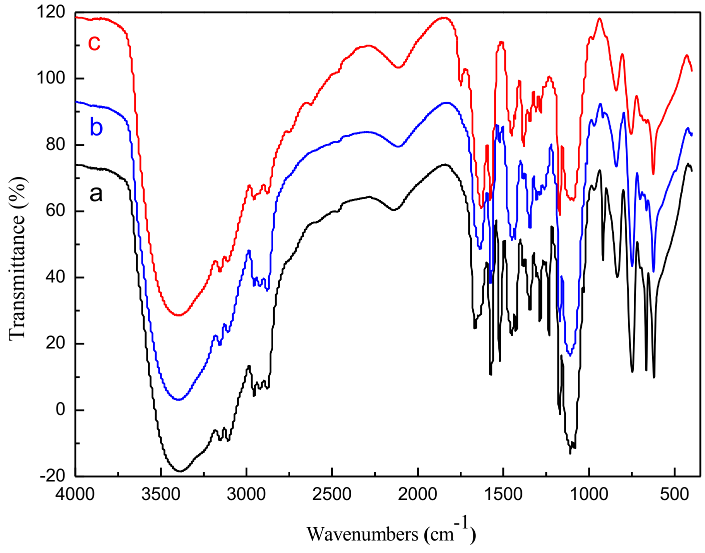

Figure S7. FT-IR spectra of three polymer ionic liquids: (a) [HO-PECH-MIM]Cl; (b) [H_2N-PECH-MIM]Cl; (c) [HOOC-PECH-MIM]Cl).

References.

[S1] Guo LY, Zhang B, Li CY, Ma XY, Chang XT, Gao X (2015) Synthesis of polyether ionic liquids and its influence on properties of phenolic resin. Plastics 44:57-59.

[S2] Guo LY, Ma XY, Li CY, Deng LL, Bai SY (2017) Preparation and catalytic properties of chloride 1-carboxyl polyether-3-mtthyl imidazole ionic liquid. Acta Petrol Sin (Pet Process Section) 33:342-348.

[S3] Guo LY, Ma XY, Wang LY, Deng LL, Jin XC (2017) Preparation and catalytic properties of chloride 1-amino polyether-3-methyl imidazole ionic liquid. Chem Ind Eng Prog 36:581-587.
